# The Influence of Visual and Olfactory Cues in Host Selection for *Bemisia tabaci* Biotype B in the Presence or Absence of *Tomato Yellow Leaf Curl Virus*

**DOI:** 10.3390/insects11020115

**Published:** 2020-02-11

**Authors:** Nicholas Johnston, Xavier Martini

**Affiliations:** Department of Entomology and Nematology, University of Florida, Quincy, FL 32351, USA; njohnston8979@ufl.edu

**Keywords:** *Bemisia tabaci*, visual, geminivirus, olfaction, TYLCV, host selection

## Abstract

The silverleaf whitefly, *Bemisia tabaci*, is one of the most destructive agricultural pests in the world, vectoring a large number of devastating viruses, including *Tomato Yellow Leaf Curl Virus* (TYLCV). When selecting a host, *B. tabaci* is primarily influenced by a range of visual and olfactory cues. Therefore, elucidating how such cues become modified in the presence of whitefly-vectored begomoviruses is critical to better understanding the epidemiology of many economically important diseases. The goal of this study was to determine how both visual and odor cues interact in the presence of TYLCV. In Y-tube olfactometer assays, whiteflies were submitted to a range of isolated visual and olfactory cues to determine behavioral changes. *B. tabaci* choices were then compared to both stimuli combined in the presence or absence of TYLCV. Under visual stimuli only, *B. tabaci* exhibited a visual attraction to the color yellow, TYLCV-infected tomato leaves, and TYLCV-infected tomato volatiles. Attraction was the strongest overall when both visual and olfactory cues from TYLCV-symptomatic tomato plants were combined, as opposed to a single isolated cue. These results highlight the importance of both sensory stimuli during *B. tabaci* host selection in the presence of an associated begomovirus.

## 1. Introduction

Silverleaf whitefly, *Bemisia tabaci* Gennadius (Hemiptera: Aleyrodidea), is a major economic pest of row crop vegetables, causing widespread feeding damage and vectoring many viruses. In tomatoes, *tomato yellow leaf curl virus* (TYLCV) is one of the world’s most devastating diseases and can result in considerable to total yield loss. Further complications arise during major growing seasons as whitefly populations become increasingly unpredictable and resistant to conventional insecticides [1]. Since TYLCV is a persistent-circulative virus, this pathogen requires 16 to 24 h to become acquired by its whitefly vector. This period of time is known as an acquisition access period [2]. The virus then requires another 17 to 24 h latent period to circulate in the whitefly before the vector can transmit TYLCV to an uninfected host. This epidemiological cycle creates a scenario where the virus is best benefited by inducing changes in the host plant phenotype that make it more attractive to the vector.

In regard to *B. tabaci* behavior when locating and selecting a host, both visual cues, such as plant color, and olfactory cues, such as plant volatiles, play critical roles. In the visible spectrum, certain wavelengths of light, such as those corresponding to the color yellow, have been shown to be more attractive to *B. tabaci*, influencing movement and host choice [3,4]. Pest management practices have taken advantage of this behavioral aspect by using methods such as yellow sticky card traps to monitor whitefly populations [5]. In theory, begomoviruses such as TYLCV may also take advantage of this behavior, since symptomatic plants exhibit yellowing of their leaves, which would further enhance the disease cycle. Since *B. tabaci* is diurnal, with peak flight activity occurring between 9:00 and 13:00 [6], it is assumed that visual cues primarily drive host selection [7]; however, host selection behavior for *B. tabaci* may be far more complex than previously thought. For instance, plant volatiles also play important roles in whitefly host selection, as evidenced by how surfactants, oils, kaolin, and other products can repel or reduce *B. tabaci* settlement on a host [8,9,10]. In addition, using volatiles as attractants or interplanting with crops such as coriander to mask the volatiles of a target crop can also prove effective [7]. Since *B. tabaci* is polyphagous, feeding on many different hosts, this insect has shown a hierarchy of host preferences when presented with different host volatiles in choice tests [11]. Though many studies have shown the attraction that *B. tabaci* exhibits towards begomovirus-induced volatiles or colors in the plant host phenotype, few to none have addressed the interactions between these different modal cues [12].

Utilizing both visual and olfactory mechanisms, TYLCV is known to affect *B. tabaci* host selection behavior, though the extent that a TYLCV-infected plant impacts each sensory mode of the vector remains to be quantified. Several past studies have shed light on the mechanisms of plant viruses increasing the attraction of an insect vector by altering the host phenotype [13]. As these viruses depend on long-term propagation via vector transmission, their success in spreading is benefited most by increasing the attractiveness of the vector’s host [12]. In addition, vector behavior can be manipulated indirectly, by increasing the attractiveness of a host’s physical characteristics, or directly, once the insect vector has fed on the infected host and acquired the virus [14]. This behavior manipulation after acquisition is an important next step since the virus depends on the vector to disperse to a new uninfected host for subsequent transmission to occur. Therefore, understanding the subtle interplay between vector-host selection and vector-virus manipulation is key to understanding the disease epidemiology and implementing the most effective management strategies.

Overall, the purpose of this study is to determine *B. tabaci* natural host selection behavior by evaluating the strength of visually attractive targets in the presence or absence of olfactory cues, in order to gauge the strength of this sensory mode alone or in combination with other cues. In addition, this study investigates how TYLCV then manipulates visual cues of the host, such as plant pigments, and olfactory cues during host selection. The behavior of whiteflies was evaluated by using a series of choice experiments between TYLCV-infected and uninfected tomato plants. A series of olfactometer tests were conducted with *B. tabaci* that determined bi-modal choice between specific visual targets. Additional olfactometer tests examined whitefly choice between visual and olfactory targets in the presence or absence of TYLCV. Finally, bioassays were conducted where *B. tabaci* was given the choice of two sensory cues combined (vision and olfaction) or a single, isolated cue.

## 2. Materials and Methods

### 2.1. Whiteflies

*B. tabaci* biotype B specimens were originally collected from infested straightneck squash, *Cucurbita pepo L.* cv. recticollis, in Quincy, Florida, in the fall of 2018. Biotype testing was conducted by genetic analysis following the protocol described in McKenzie et al. [15]. Whiteflies were reared on collard—*Brassica oleracea*—in whitefly-proof screen cages in a rearing chamber (26 ± 2 °C) for at least five generations. Rearing on collard was primarily conducted to produce a TYLCV-free colony since *B. oleracea* is a non-host and does not serve as a reservoir for the virus. Sentinel tomato plants present in the TYLCV-free whitefly colony were not infected by TYLCV during the whole duration of the experiment. It has been demonstrated that whitefly host preference is affected by past feeding experience [16]; however, switching the whitefly’s host from collard to tomato in Y-tube assays was designed to examine *B. tabaci* choice, despite past host feeding bias and under a host-switching scenario. Subsequently, olfactometer and closed-cage assays used only adults—regardless of age—taken from laboratory-maintained colonies. While both sexes were used, the sex of each whitefly was recorded in each Y-tube olfactometer experiment to confirm that host selection was not biased towards one sex only.

### 2.2. Tomatoes and TYLCV Infection

Hybrid tomatoes—*Solanum lycopersicum*—of the Florida 47 R variety were utilized in all described experiments. Tomato seeds were treated with thiram and planted on plastic trays (51 × 25 cm) containing agricultural substrate (Jolly Gardener Proline C/B Growing Mix). Seedlings were then transplanted into 11.5 cm^2^ black nursery pots (one seedling per pot) 3 weeks after sowing and maintained under greenhouse conditions.

After 1 month, tomato plants were moved to white, mesh screen cages (40 × 40 × 60 cm) and inoculated with TYLCV. Plants were inoculated by introducing 100 adult *B. tabaci* collected from TYLCV-symptomatic tomatoes in the field to each cage. Whiteflies were then removed 14 days later. After an additional month past the initial date of inoculation, plants were tested for TYLCV by RT-PCR. DNA extraction was performed with the Genesee Sci. Quick-DNA Plant/Seed Miniprep Kit (Cat #: 11-391). PCR reactions were prepared using GoTaq Hot Start Master Mix (Cat #:MF122). Begomovirus degenerate primers AV494 (5′GCC Y AT RTA YAG RAA GCC MAG-3′) and AC1048 (5′GGR TTD GAR GCA TGH GTA CAT G-3′) were used for amplification. Amplification conditions consisted of 2 min denaturation at 94 °C, followed by 35 cycles of 95 °C for 1 min, 54 °C for 45 s, and 72 °C for 1 min, with a final extension at 72 °C for 10 min. Amplicons were sent to the Florida State University sequencing facility for sequencing, and contained a 100% identity to TYLCV. Control plants were confirmed to be uninfected by TYLCV with the same RT-PCR test. The tomato leaves used in olfactometer experiments were collected two months following inoculation.

### 2.3. Experiment 1 Preference of B. Tabaci for Visual Cues Only

Split-tube, choice bioassays were conducted to determine the degree of attraction that *B. tabaci* displayed to a multitude of visual cues. The experimental setup consisted of a glass Y-tube positioned horizontally under a fluorescent light source mounted within a white fiberboard box for uniform light diffusion. Visual targets were then placed underneath each arm of the glass Y-tube, and a single whitefly was released at the bottom of the tube. Each whitefly was given 15 min to choose between the two arms, and all visual targets were replaced and switched to opposite sides after every two whitefly choices, to reduce the influence of potential directional bias in the glassware (a preliminary test was run to assess that there was no bias between the right and left arm of the olfactometer). The visual targets included (1) one 3 × 1 cm rectangular slip of yellow construction paper, (2) one control tomato leaf (uninfected), and 3) one symptomatic TYLCV-infected tomato leaf. Using these visual targets, the study consisted of a five-treatment set up, as follows: (1) yellow color vs. no visual target; (2) control tomato leaf vs. no visual target; (3) TYLCV-infected tomato leaf vs. no visual target; (4) TYLCV-infected tomato leaf vs. control tomato leaf; and (5) yellow color vs. TYLCV-infected tomato leaf. Five trials of 10 individual whiteflies were conducted per treatment (total of 50 whiteflies per treatment).

### 2.4. Experiment 2 Preference of B. Tabaci for Visual Cues Combined with Odor Cues

**Figure 1:**
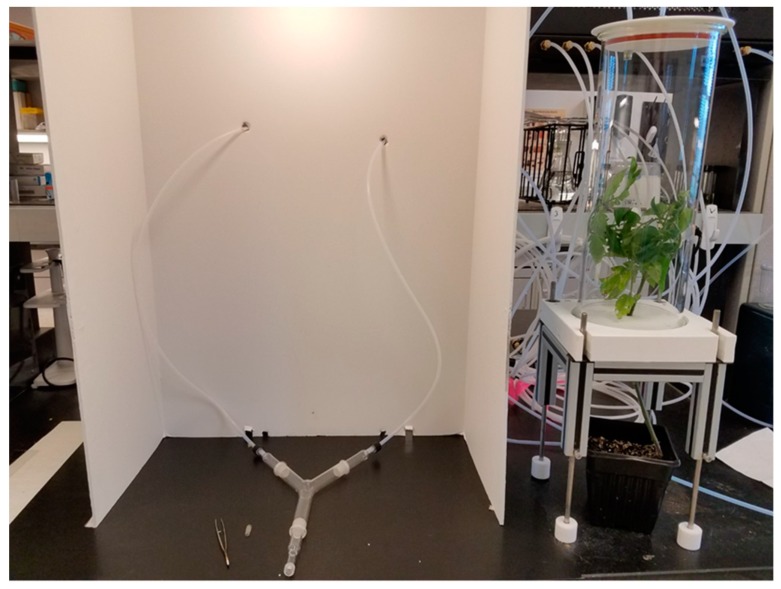
Y-tube olfactometer setup. Volatiles from a tomato plant (pictured right) were blown through a single arm of the glassware placed inside a white, fiberboard box with a uniform light distribution (pictured left).

To develop a more complete picture of how sensory stimuli influence whitefly behavior, an olfactory component was introduced, in conjunction with several visual cues used in the first study. Differences in attraction between volatiles emitted by control tomato and TYLCV-infected tomato were also evaluated. The odor source consisted of a single tomato plant either TYLCV-infected or uninfected. Tomato plants were positioned within a cylindrical glass jar (16 × 50 cm) sealed with a Teflon lid (Sigma Scientific, Gainesville, FL, USA). Two outlets were positioned on the Teflon lid, with Teflon tubing connecting one outlet to the olfactometer and the other outlet to a single arm of the Y-tube glassware. For treatments that required one Y-tube arm to receive clean air, Teflon tubing was connected directly from the olfactometer to the Y-tube. Glass jars received charcoal-purified and humidified air at 0.1 L/min from a custom-made air delivery system (Sigma Scientific, Gainesville FL, USA). The Y-tube glassware was positioned horizontally under a fluorescent light source mounted within a white fiberboard box for uniform light diffusion and the removal of any extraneous visual stimuli (Figure 1). Whitefly adults were released individually at the base of the Y-tube and were given 15 min to exhibit a behavioral response. A positive response was recorded when an insect moved from the glass tube stem and moved 1 cm into either arm of the Y-tube. Combined visual and olfactory trials consisted of the six treatments outlined in Table 1. The test conditions and number of replicates were similar to those employed in Experiment 1.

### 2.5. Statistical Analysis

After normal distribution and equal variance checks, a two-tailed paired t-test was conducted in R (R 3.5.1, R Foundation for Statistical Computing, Vienna, Austria) to determine the significance of whitefly host selection between visual and/or olfactory targets for each treatment. Additional paired t-tests were run solely on whiteflies of each sex in each treatment to rule out sex bias correlated with whitefly choice. For TYLCV treatments combining visual and olfactory cues, the number of *B. tabaci* individuals’ selections of either one cue or both cues combined was summed and reported as a proportion of the sample size for each treatment, where n = 50.

## 3. Results

### 3.1. Experiment 1 Preference of B. Tabaci for Visual Cues Only

**Figure 2:**
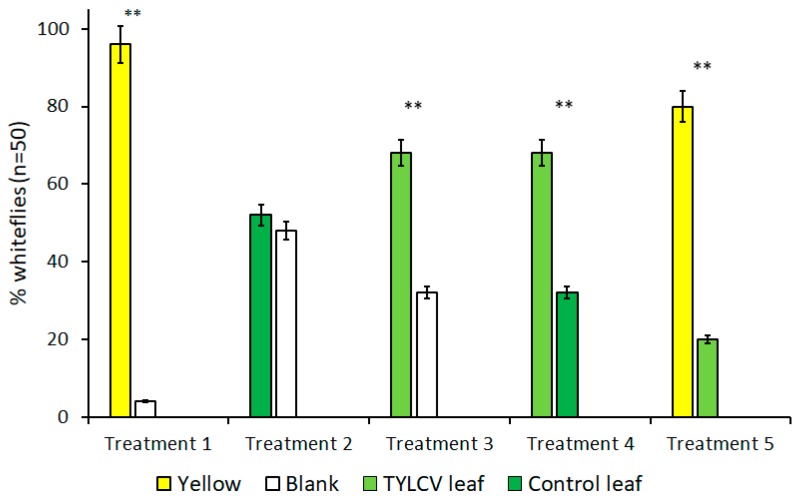
Attraction of *Bemisia tabaci* to visual targets in dual-choice Y-tube assays. Each bar represents a unique visual target placed underneath an arm of Y-tube glassware. Treatments where *B. tabaci* significantly selected one visual cue over the other are indicated by **, where α = 0.01.

The results of all trials performed with visual cues alone are summarized in Figure 2. Due to *B. tabaci*’s attraction to the color yellow, treatments that included yellow targets served as both a positive control and to gauge the attraction level of other visual targets in the presence of a known attractant. As expected, for all treatments where yellow visual targets were included, *B. tabaci* exhibited a preference for the yellow target over alternative choices, including a TYLCV-infected tomato leaf (t = 4.74, df = 4, *p* < 0.009) and clean air (t = 18.78, df = 4, *p* < 0.001).

In the absence of yellow visual targets, however, whitefly behavior changed drastically. Despite showing a preference for the color yellow over a TYLCV-symptomatic leaf in previous trials, *B. tabaci* preferred the visual stimulus of a TYLCV-symptomatic leaf over clean air (t = 4.81, df = 4, *p* = 0.009), which is a behavior more significant in light of *B. tabaci’s* lack of preference between a healthy tomato leaf and clean air that served as control treatment (t = 1, df = 4, *p* = 0.37). Finally, *B. tabaci* showed a direct preference for the visual cues of a TYLCV-symptomatic tomato leaf compared to a healthy tomato leaf (t = 4.81, df = 4, *p* = 0.009). Comparisons of these treatments illustrate how visible phenotype alterations alone caused by TYLCV can, in turn, alter the behavior of *B. tabaci* and attract the vector to infected hosts.

### 3.2. Experiment 2 Preference of B. Tabaci for Visual Cues Combined with Odor Cues

**Figure 3:**
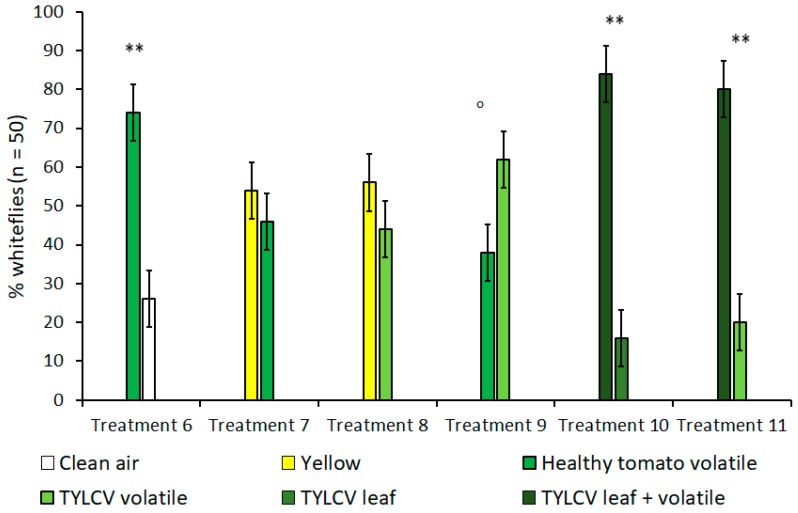
Attraction of *Bemisia tabaci* to either visual/olfactory cues or a combination of both in dual-choice Y-tube assays. Treatments 5 and 6 compare the combination of visual and olfactory cues from a *tomato yellow leaf curl virus* (TYLCV) tomato to a single, isolated cue. Treatments where *B. tabaci* significantly selected one cue over the other are indicated by ‘o’ where α = 0.1 and ** where α = 0.01.

In olfactometer experiments, when given a choice between a yellow visual target without an accompanying olfactory cue and the volatile of a tomato leaf without a clear, visual target, *B. tabaci* showed no preference for one over the other (Figure 3). This lack of preference remained true for both healthy tomato leaves (t = 1.63, df = 4, *p* = 0.18) and TYLCV-symptomatic tomato leaves (t = 1.50, df = 4, *p* = 0.21); a fact underscored by the high preference for yellow targets in the previously described “visual cues only” experiments. Subsequent control experiments that compared *B. tabaci*’s choice between healthy tomato volatiles and clean air also showed a significant preference for tomato volatiles (t = 11.5, df = 4, *p* < 0.001) when no visual targets were present. In additional treatments that looked at *B. tabaci* selection with volatiles only, whiteflies showed a slight attraction to the volatiles of a TYLCV-infected tomato plant over the volatiles emitted by a healthy tomato; however, this attraction was still under the level of significance of α = 0.05 (t = 2.45, df = 4, *p* = 0.070).

Since isolated visual and olfactory cues from TYLCV-infected tomato were shown to increase *B. tabaci* attraction in previous treatments, treatments 10 and 11 were designed to investigate whether combining both visual and olfactory cues was more attractive to *B. tabaci* than either stimulus alone and to what degree (Table 1, Figure 3). In both TYLCV treatments, *B. tabaci* overwhelmingly preferred the combination of visual and olfactory stimuli compared to an isolated visual target (t = 6.67, df = 4, *p* = 0.003)—a TYLCV-symptomatic leaf—and an isolated olfactory target (t = 9.49, df = 4, *p* < 0.001)—a TYLCV/tomato volatile emission. For both experiment 1 and 2, whitefly preference was not biased towards sex or Y-tube position, regardless of treatment.

## 4. Discussions

TYLCV is one of many begomoviruses that can potentially alter the phenotypic and physiological traits of its host to increase the attraction of the insect vector [17]. This increased attraction of *B. tabaci* has severe consequences regarding TYLCV epidemics, such as increasing the proportion of infected vectors in the environment and speeding up the spread of the disease [18,19]. As TYLCV is a persistent virus, alterations in infected host plants have also been shown to increase *B. tabaci* fitness to the detriment of the host [20], further emphasizing the need to understand how the viral manipulation of host phenotypes influences vector behavior. In this study, treatments combined and contrasted visual targets, such as a yellow color, with tomato volatiles, in order to gauge the strength of each sensory stimulus during host selection. As whiteflies use a combination of both visual and olfactory cues during host location and selection, determining the strength of each stimulus under different conditions in the presence or absence of begomovirus infection was the primary goal of this research. A similar study conducted by Fereres et al. in 2016 examined how related begomoviruses, including *tomato chlorosis virus* (ToCV) and *tomato sever rugose virus* (ToSRV), vectored by *B. tabaci*, modify the host phenotype, leading to a modification in vector behavior [21]. This study found that ToCV increased the attractiveness of the plant host’s visual cues, while olfactory cues had no effect on choice, even decreasing the attractiveness to non-viruliferous whiteflies. Unlike ToCV, the inoculation of tomato with ToSRV increased the attractiveness of tomato volatiles to *B. tabaci*, illustrating how even closely related viruses can affect the same vector in different ways. Understanding these factors will shed additional light on viral-vector–host interactions for the most economically important diseases of tomatoes worldwide.

In this study, bioassays that only tested visual cues revealed several key aspects of *B. tabaci* behavior and served to underscore many of the results of treatments that combined visual and olfactory cues. Whiteflies, and other related insects in the suborder Sternorryhncha, are diurnal and are known to use visual cues as a predominant stimulus during dispersal and host selection [3,22]. Furthermore, wavelengths of light corresponding to the color yellow have been proven to be the most attractive visual target for *B. tabaci*, as shown from past studies and abundant catches in yellow, sticky card traps [5,23]. Treatments that contained yellow visual targets, unsurprisingly, had higher numbers of whiteflies selecting these targets. Unexpectedly, *B. tabaci* did not show a preference for healthy (green) tomato leaves, even in the absence of other visual targets. *B. tabaci* exhibiting a preference for TYLCV-symptomatic leaves over clean air, yet showing a preference for the color yellow in the presence of TYLCV leaves, demonstrates how a spectrum of visual attractiveness exists that is potentially based on the amount of specific yellow wavelengths reflected. Preliminary data and past studies provide further evidence in support of *B. tabaci*’s spectrum of attractance, where whiteflies showed a high attraction to the color yellow, lower attraction to the color green, and no attraction to the color red [4]. Finally, the visual attraction of *B. tabaci* to TYLCV-symptomatic leaves over healthy leaves partly explains how a virus-induced change in its host’s phenotype can alter the vector behavior, which, in turn, can promote the spread of the virus.

In light of the behaviors *B. tabaci* exhibited in vision-only bioassays, many insights were made from adding an additional olfactory stimulus to determine how this would alter whitefly behavior in the presence of a known visual attractant (i.e., yellow color). Setting olfactory cues from both healthy and TYLCV-infected tomato in direct opposition to yellow visual targets completely neutralized the significant attraction to yellow seen in previous experiments. Unlike green leaf vs. blank (treatment 2) experiments using only visual cues, green leaf vs. blank experiments using only volatile cues (treatment 6) revealed that *B. tabaci* was significantly attracted to healthy tomato volatiles in the absence of other interfering cues (Figure 2 and Figure 3). This finding supports the theory that volatiles may influence *B. tabaci* host selection to a greater degree than visual cues. In any case, both visual and olfactory cues combined were unequivocally stronger than either stimulus alone with regards to *B. tabaci* host selection, as seen in treatments 5 and 6 (Figure 3). Though there was no difference between the isolated cues themselves, the results from these treatments clearly illustrate how the combination of multiple cues can have an additive effect on *B. tabaci* host selection, especially in the presence of whitefly-vectored diseases such as TYLCV.

**Figure 4:**
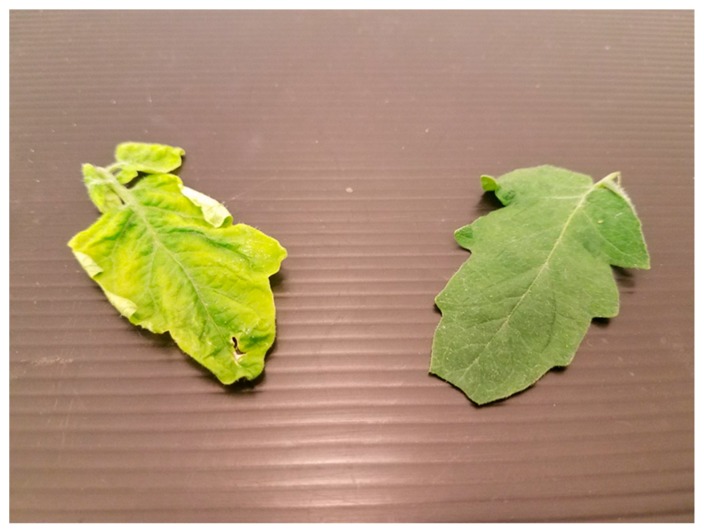
Visual differences between a healthy tomato leaf (pictured right) and a TYLCV-symptomatic tomato leaf (pictured left) removed 2 months after inoculation.

Despite several insights being revealed from the experiments in this study, there are many additional avenues of research to be conducted in this field. Since all TYLCV-infected tomato plants used in these bioassays were over 2 months past the date of initial inoculation, more results might be gained by examining how *B. tabaci*’s visual and/or olfactory attraction increases incrementally over time, in conjunction with the development of TYLCV symptoms, especially since whiteflies showed no significant visual attraction to a healthy tomato. Even with naked eyes, a clear difference can be seen in the coloration of a healthy tomato leaf and a TYLCV-symptomatic leaf 2 months past inoculation (Figure 4). A more time-sensitive, qualitative study could further reveal how TYLCV manipulates *B. tabaci* by increasing visual attraction to the host plant over time. In addition, similar or repeated experiments using whiteflies reared on both healthy and TYLCV-infected tomato rather than collard would prove useful, since *B. tabaci* has been shown to prefer the host plant on which it developed in past studies [24]. Determining how *B. tabaci* host selection is influenced by nymphal development on the same host, especially one infected by a virus, could also yield important data regarding how vector acquisition of the virus subsequently alters vector behavior.

## 5. Conclusions

Though past research has investigated the visual and olfactory mechanisms of *B. tabaci* host selection, no studies, to the authors’ knowledge, have compared the relative strength of each stimulus, and how the behavior linked to the strength of each stimulus changes in the presence of TYLCV. Most *B. tabaci* control programs emphasize the use of either visual control methods, such as UV-reflective mulch and sticky card traps, or olfactory control methods, such as natural plant-based repellents and intercropping; however, tactics that target both vision and olfaction are needed for the most effective control [7,25]. This study has shown how *B. tabaci* host selection is complex and how multiple stimuli are utilized to varying degrees. It was also found that the presence or absence of a begomovirus such as TYLCV can alter pre-existing host selection behavior. Not only will future research regarding the mechanism of *B. tabaci* host selection lead to an improved cost/benefit analysis in determining more efficient management tactics, but it will also contribute to a better understanding of whitefly vector behavior and epidemiology concerning associated and economically important diseases.

## Figures and Tables

**Table 1:** Experimental setup of six treatments with isolated or combined visual and olfactory cues. Visual or olfactory cues were switched between arms 1 and 2 after every two choices made by individual whiteflies.

Treatment	Arm 1 of Y-Tube	Arm 2 of Y-Tube
6	Blank/clean air	Volatiles from healthy tomato
7	Yellow visual target	Volatiles from healthy tomato
8	Yellow visual target	Volatiles from TYLCV-infected tomato
9	Volatiles from healthy tomato	Volatiles from TYLCV-infected tomato
10	TYLCV-leaf visual target	TYLCV-leaf visual target, Volatiles from TYLCV-infected tomato
11	Volatiles from TYLCV-infected tomato	Volatiles from TYLCV-infected tomato, TYLCV-leaf visual target

## Data Availability

All data was ultimately collected through funding provided by the University of Florida and is, therefore, UF property. The data are available upon request.
